# Editorialets

**Published:** 1907-07

**Authors:** 


### Editorialets.

THIS issue begins the twenty-third year of a pretty lively existence for the "Red Back." Congratulations and subscriptions are in order.

CHANGE OF BASE, for greener fields and pastures new. We regret the loss to Austin of our esteemed friend and colleague, Dr. M. M. Smith, of the *Texas Medical News.* He has removed, with his family and his journal, to Dallas. Dr. Smith, having been appointed medical director of the Order of Praetorians, found it advantageous to make Dallas his headquarters. He will find there, too, wider opportunity for the exercise of his talents, a better field for the publication of the *News,* for the practice of medicine,—and, I rather suspect, that a position in one of the medical colleges will be offered him. He is qualified to fill any chair, but perhaps would shine more brilliantly in sanitary science or public hygiene, a branch with which he has practical acquaintance, and has been long identified. Our loss is Dallas' gain; and, while we regret to lose him, he carries with him the high esteem and warm regard of hosts of attached friends—none of whom love him more or more sincerely wish him every success than his old friend of the "Red Back."

THE NEW MEDICAL PRACTICE ACT, the "One Board Bill," went into effect July 12th inst. At the time of our going to press the

Governor has not appointed the board. I will give the names next issue.

---

The following is a verbatim copy of the first report made to Postmaster General Cortelyou by a newly-appointed postmaster in a rural district of North Carolina:

"muster Jorge Cortelyou, President of the United States,—Dear sir been required by the instrucions of the post office to report quarterly, I now fulfill that plesent duty by reporting as follers. The harvestin has been going on purty wel and most of the naburs have got thur cuttin about dun, wheet is hardly a average crop on rollin lans corn is yellerish and wont cut morn ten booshils to the aker the health of the communty is only torrerable messels and cholry has broken out in abought 2 and a half mile from hear, thar are a powful awaken on the subject of religion in the Potts naburhood and many soles are bein made to know thar sins forgiven. Miss nancy Micks a neer nabur har a new baby but he is a poor scraggy little feller and wont live half his day this is about, all i know and have to report the present quarter give my respects to MISS Cortelyou and subscribe myself yours trooly."—*Harper's Weekly.*

---

Kitty Dixon is the name of a charming little story of love and war,—the post-bellum blending of the Gray and the Blue,—by the talented editor of the *Southern Clinic,* our friend, Dr. C. A. Bryce. The scene is laid in Old Virginia, and the chief characters are two colonels—one on each side, and their son and daughter (Kitty Dixon), respectively. It reminds me of Pages' On Newfound river, an "Ole Viginy" story. The pretty little pictures are by Dr. Bryce's talented daughter, Miss Mildred Bryce. The little volume is exquisitely bound in blue and gray and gilt, and will make an appropriate memento of the Jamestown Exposition now being held on the historic soil of the mother State. The price is 50 cents. Dr. Bryce is to be congratulated on the dainty and sweet little story. He calls it "A wee bit of love and war."

---

Bills have been sent to all subscribers in arrears. I will appreciate a remittance. If any subscriber should receive a sample copy, let him hand it to a friend and solicit his subscription, please.

---

Another Editor Honored.—I am much gratified to note that at the recent meeting of the Louisiana State Medical Association our esteemed colleague of the *Medical Recorder* (Shreveport), Dr.

Oscar Dowling, was elected President of the Association. The Association has honored itself and is to be congratulated.

---

SUBSTITUTION KNOCKED OUT IN NEW YORK.—The Page bill making substitution a penal offense passed the New York Legislature after a bitter fight against it by some of the large drug concerns, who are notorious substitutors and imitators. The credit for the passage of this much-needed measure is largely due to Charles Roome Parmele of the Parmele Pharmaceutical Company New York. I will publish it in next issue with some "remarks." We congratulate the New York profession and Mr. Parmele on the passage of this righteous act.

---

A MEDICAL NIGHT SCHOOL has been chartered in St. Louis. It is called The Hippocratean College of Medicine. I notice amongst the faculty Drs. E. Lanphear, Chambers, Feigenbaum, S. S. Kohn, Ohmann-Dumesnil, and A. H. Koch. Sessions will be held five evenings in the week, and three or four hours Saturday afternoons will be given to clinical work. The school is for the benefit of men who are studying medicine but have to work meantime.

---

DR. VARD H. HULEN, of Houston, has returned to San Francisco, Cal., to accept charge of the eye department in the University of California.

---

THE *Interstate Medical Journal* (St. Louis) announces the purchase of the *St. Louis Courier of Medicine,* one of the oldest medical journals in the West, and its consolidation with the *Interstate* on July 1st.

---

WHEN SHOCKED BY ELECTRICITY.—The following is clipped from the Chicago *News*. It gives some interesting and useful information concerning electrical shocks:

In addition to artificial respiration the use of atropine, hypodermically, as a respiratory stimulant, will suggest itself, with glonoin and strychnine as circulatory stimulants. The clipping follows:

"How many in this twentieth century are murdered through ignorance? I allude to people shocked by electricity. When a man is electrocuted he receives a shock of over 2000 volts. And under the best conditions I will assert that if the condemned should receive a pardon after receiving this charge of 2000 volts in ten minutes' time he might be alive and enjoying the benefit of that pardon.

"Trolley wires which kill so many people carry only 500 volts —a force just sufficient to give a very interesting vaudeville exhibition, showing how a person is revived after being shocked.

"Every fireman and policeman in the city ought to know what to do when a person is shocked by electricity. When a man is under the influence of ether he breathes naturally, but when 'under the influence' of electricity he does not breathe at all. The first thing to do after a man has been shocked is to force him to breathe. Take off the coat, place it under the shoulders. Open the clothes around the throat and chest. Draw the arms over the head and out to full length. This expands the chest and permits the air to enter. Bring the arms back, doubled at elbows, and bring them forcibly against the chest, expelling the air. Keep this up for one hour if the person is not revived sooner, and breathes as fast as a person should breathe. While doing this send some one for a doctor.

"A young man of South Bend received a shock of 2500 volts. He had hardly fallen to the floor before he was grabbed by an electrician who tried to revive him. In five minutes' time he was all right. About twenty-four hours later he felt pain from the shock. He suffered for about a week and then was as well as ever.

"A man who revives a person shocked by electricity is as much a hero as one who saves another from a burning building."—*Ernest Filer, in Hot Springs Medical Journal.*

